# SPARC Controls Melanoma Cell Plasticity through Rac1

**DOI:** 10.1371/journal.pone.0134714

**Published:** 2015-08-06

**Authors:** Edgardo Salvatierra, Mariano J. Alvarez, Claudia C. Leishman, Elvia Rivas Baquero, Viviana P. Lutzky, H. Eduardo Chuluyan, Osvaldo L. Podhajcer

**Affiliations:** 1 Laboratory of Molecular and Cellular Therapy, Instituto Leloir-CONICET, Buenos Aires, C1405, Argentina; 2 Laboratory of Immunomodulators, School of Medicine, Centro de Estudios Farmacológicos y Botánicos (CEFYBO), Consejo Nacional de Investigaciones Científicas y Tecnológicas (CONICET)-University of Buenos Aires, Buenos Aires, Argentina; Casey Eye Institute, UNITED STATES

## Abstract

Cell transition to a more aggressive mesenchymal-like phenotype is a hallmark of cancer progression that involves different steps and requires tightly regulated cell plasticity. SPARC (Secreted Protein Acidic and Rich in Cysteine) is a matricellular protein that promotes this transition in various malignant cell types, including melanoma cells. We found that suppression of SPARC expression in human melanoma cells compromised cell migration, adhesion, cytoskeleton structure, and cell size. These changes involved the Akt/mTOR pathway. Re-expression of SPARC or protein addition restored all the cell features. Suppression of SPARC expression was associated with increased Rac1-GTP levels and its membrane localization. Expression of the dominant negative mutant of Rac1 counteracted almost all the changes observed in SPARC-deficient cells. Overall, these data suggest that most of the SPARC-mediated effects occurred mainly through the blockade of Rac1 activity.

## Introduction

One of the hallmarks of epithelial cancer progression is the transition to a more aggressive mesenchymal phenotype. During this process, cells adopt migratory attributes, change their cell adhesion properties, polarity, and reorganize actin cytoskeleton, facilitating their dissemination away from the primary tumor [1]. These malignant cells may settle in a new environment to generate metastatic foci where they reduce their motility and establish interactions with new neighbors and matrices, experiencing a reversion back to an epithelial phenotype. These transitions require from cancer cells to have the plasticity and capacity to adapt to different environments.

SPARC is a highly conserved matricellular glycoprotein whose expression has been associated with aggressive, mesenchymal-like phenotypes in a variety of human cancers, including melanoma [2]. Indeed, previous studies have demonstrated that the inhibition of SPARC expression abrogated the tumorigenicity and metastatic dissemination of cancer cells in melanoma [3–6] and glioma human xenografts tumors in nude mice [7]. Current knowledge obtained mainly with endothelial cells indicates that SPARC regulates cell shape by inhibiting cell spreading [8, 9], followed by changes in actin stress fibers architecture, and focal adhesion disassembly [10]. Thus, essential traits of the transition to a mesenchymal phenotype seem to be controlled in part by SPARC, although the potential mediators and mechanisms underlying this control remain unclear.

The intracellular pathways triggered by SPARC have only been partially described. For example, SPARC-driven glioma cell survival and invasive capacity have been associated with increased activities of FAK and ILK kinases [11] involving the phosphatidylinositol 3-kinase (PI3K)-Akt axis [12]. Activation of the PI3K/Akt pathway by SPARC promotes melanoma cell invasion and survival advantages [13–15] linked to a worse prognosis [16, 17]. SPARC-mediated melanoma cell migratory capacity is SLUG dependent [14], while the transendothelial migration capacity of melanoma cells is associated with SPARC-driven E- to N-cadherin switching [18]. Thus, essential traits of the transition to a mesenchymal phenotype seem to be controlled in part by SPARC, although the potential mediators and mechanisms underlying this control remain unclear.

In this study we aimed to unravel a potential intracellular mechanism of action of SPARC that would help explain its diverse roles, focusing on human melanoma cells for which the role of SPARC as a pro-tumorigenic and pro-mesenchymal protein has been conclusively demonstrated [2, 19, 20]. The present data show that SPARC modulates different features of melanoma cell aggressiveness such as cytoskeleton architecture, cell size, and migration. We demonstrate that the sGTPase Rac1 acts as an intracellular mediator of SPARC effects since blocking Rac1 activity restored most of the cell phenotype changes induced by the suppression of SPARC expression.

## Materials and Methods

### Reagents

Integrin expression was assessed by flow cytometry using CD49a-phycoerythrin (CD49a-PE), CD49b-PE, CD49c-PE, CD49d-PE, CD49e-PE, CD49f-PE, CD29-allophycocyanin (APC) monoclonal antibodies (Pharmingen, San Diego, CA, USA) following manufacturer’s instructions. Nonspecific IgG of the corresponding class were used as isotype controls. ECM proteins fibronectin, from human plasma, collagen type IV, laminin, and vitronectin were from Sigma (St Louis, MO, USA). Matrigel was from BD Biosciences (San Jose, CA, USA). Native SPARC was purified from A375N human melanoma cells conditioned media.

### Vectors

The human SPARC-coding sequence was obtained by PCR from A375 cDNA and cloned into HindIII/ApaI sites of pcDNA6/V5-HisB (Invitrogen, Carlsbad, CA, USA). pcDNA6-SP is a V5/6His tagged human SPARC expression vector driven by the CMV promoter. Empty plasmid pcDNA6/V5-HisB was used as a control. Adenoviral vectors carrying SPARC and β-galactosidase genes (AdSP and Adβgal) were obtained as described [4]. Plasmids coding for wild and mutant versions of the RHO family sGTPases and Rac1-GFP chimeric have been already described [21, 22].

### Cell transduction

Cells were grown up to 80% confluence in monolayers and transduced with 5x10^8^ TCID50/ml of the different adenoviral vectors for 6 hours. In the final step, the transduction medium was replaced with fresh complete medium; cells were incubated for an additional 20 hours, trypsinized, counted, and used. Transduction efficiency was assessed 36 hours after transduction with Ad-bgal followed by X-gal [23]. Experiments were run only when transduction efficiency was better than 75%. Lentiviral vectors expressing SPARC were constructed by cloning by RT-PCR the SPARC sequence from A375 RNA into pEntry/D-TOPO followed by Gateway recombination in pLenti4/TO/V5 following the manufacturer's instructions (Invitrogen, La Jolla, CA, USA). Enhanced green fluorescent protein (eGFP) was used as a control. Lentiviral stocks were produced following transfection of lentiviral plasmids with Virapower mix (Invitrogen, Carlsbad, CA, USA) into 293T cells. Forty-eight hours collected supernatants were used to infect pancreatic cancer cell lines.

### Cell culture

IIB-MEL-LES and A375N human melanoma-derived cells were grown in melanoma media. Pancreatic cancer cell lines, MiaPaca2 and Panc1 were grown in DMEM high glucose. All media were supplemented with 10% FBS (Natocor, Carlos Paz, Cordoba, Argentina) and antibiotics. Cell cultures were maintained at 37°C and 5% CO_2_ in a humidified incubator. Established clones were thawed from original stocks, selected in G418, and routinely checked for SPARC production and mycoplasma absence. SPARC expression plasmid pcDNA6-SP or control pcDNA6 were transfected in L1D cells with Lipofectamine 2000 (Invitrogen, Carlsbad, CA, USA). Stable transfectants (L1Dpc6 y L1DpcSP) were selected in 5 μg/ml Blasticidine-HCl (Invivogen, San Diego, CA, USA). Plasmids encoding for sGTPases mutants were transfected as described above and cells were used 24h later for immunofluorescence and F-actin staining.

### Cell adhesion and spreading

Cells were seeded in flat-bottom 96-well plates (Corning-Costar, Wilkes Barre, PA, USA) or 12 mm glass coverslips coated with 10% FBS containing medium or with ECM proteins (30 μg/ml fibronectin, 30 μg/ml collagen type IV, 10 μg/ml laminin, 10 μg/ml collagen type I, or 150 μg/ml Matrigel, washed with PBS and blocked with 5 mg/ml BSA containing culture medium). Subconfluent cells were detached with 1.25 mM EDTA in PBS, washed with medium, and incubated for 30 min with 10 μg/ml of the selected antibodies. At the end, cells were added to each well, and allowed to adhere for 45 min at 37°C in 5% CO_2_. Non-adherent cells were removed by washing with Ca^2+^ and Mg^2+^—containing PBS. Remaining adherent cells were assessed by CellTiter 96 AQueous One Solution Cell Proliferation Assay (Promega, Madison, WI, USA).

### Cell migration assays

Cells diluted in DMEM/F12 were seeded (15.000/well) in 8-um pore 10μg/ml fibronectin or collagen type I-coated membranes of AP48-well Boyden chambers (Neuro Probe Inc., Gaithersburg, MD, USA) with 10% FBS supplemented DMEM/F12 media in lower chamber. After 4h, membranes were fixed for 5 min in cold methanol or 4% paraformaldehyde (PFA) and stained with 1μg/ml Hoechst. Non-migrated cells located on the upper surface were scrapped and migrated cells were registered using fluorescent microscopy at 20X magnification. Migrated cells were automatically counted in each image using CellProfiler homemade pipeline [24]. Each treatment was assayed twice in triplicate.

In scratch assays melanoma cells were grown to confluence in 12-well plates (Costar Corning, Inc, Corning, NY) in complete medium. After starving cells for 16 hours a 200-μL pipette tip was used to perform two crossed scratches in the confluent monolayer followed by medium replacement with fresh medium. Sixteen hours after the scratch images were acquired (using the center of the cross for orientation), with an Olympus 1X71 microscope and an Olympus DP72 camera and DPController Software (Olympus America, Inc; Center Valley, PA). Measurements of scratch area were performed using Image J (NIH, http://rsb.info.nih.gov/ij/). Three scratches were used for each treatment condition and experiment. Rates of scratch closure were calculated as the closed area (pixel) by cells relative to the initial scratch.

### F-actin visualization

Cells were stained with Alexa594-phalloidin (Invitrogen, Carlsbad, CA, USA) in 5 mg/ml BSA-containing PBS. Samples were washed with 0.01% Tween-20-containing PBS. Slides mounted with Fluorsave reagent (EMD Chemicals, San Diego, California) were visualized with an optical microscope (plan apo 60xA/1.4 and plan fluor 100x/1.3 objectives, Eclipse 6600, Nikon, Amsterdam, Netherlands), or evaluated by confocal microscopy (C-apochromat 63x/1.2 objetive, LSM 510 META, Carl Zeiss, Jena, Germany). For sGTPases imaging studies, cells were detached with EDTA 24 hours after transfection and plated on coverslips with complete medium for 90 min, and fixed and stained for F-actin.

### Focal adhesion distribution

Cells were grown for 48 hours on 12 mm coverslips coated with ECM proteins in 10% FBS containing medium, washed once with 37°C pre-warmed medium, fixed in 1% PFA/PBS and permeabilized with 0.2% triton-X100 in PBS, F-actin and phosphor-tyrosine stained. Area and relative localization were assessed using CellProfiler software.

### Quantitative RT-PCR

mRNA expression levels were assessed using qRT-PCR. ACTB and TBP were used as internal controls for normalization. One μg of RNA was reverse-transcribed using the AffinityScript qRT-PCR cDNA Synthesis Kit (Stratagene, La Jolla, CA, USA). Specific primers for ACTB, TBP[25], ITGA6, and ITGB1 transcripts were designed and the Brilliant II SYBR Green qRT-PCR Master Mix (Stratagene, La Jolla, CA, USA) was used according to the manufacturer’s instructions. Reactions were quantified with a real-time thermocycler Mx3000p (Stratagene, La Jolla, CA, USA). Relative quantification was performed using MxPRO software (Stratagene, La Jolla, CA, USA). Sequence of Primers: ITGA6se:TTGTTGCTACTGGCTGTTTTG, ITGA6as:TCCCTTTCTTGTTCTTCTTGA; ITB1se:CAAAGGAACAGCAGAGAAGC,ITGB1as:ATTGAGTAAGACAGGTCCATAAGG.

### Sequencing Rac1 in cell lines

PCR primers (Rac1AS: TTACAACAGCAGGCATTTTC, Rac1SE: ATGCAGGCCATCAAGTGTGT) were designed for amplification of full coding region (CDS) of Rac1 and Rac1b variant (GID 5879), and used to amplify cDNA of each melanoma cell line. The amplicon were DNA sequenced using Big dye terminator kit v3 in ABI PRISM 377 DNA Sequencer (Applied Biosystems, Foster City, CA, USA) and compared to GenBank deposited sequence of human Rac1 gene (GID: 5879).

### Determination of cell volume and protein concentration

To compute cell volume, we measured trypsinized cells as spheres using diameter parameter from cell counter Countess (Invitrogen, Carlsbad, CA, USA). Total protein of known cell number was measured by Bradford assay (Bio-Rad, Hercules, CA, USA) and protein concentration was estimated as ratio the of protein mass to cell volume. Protein and cell volume determinations were estimated in five independent assays for statistical analysis of comparisons.

### Small GTPase activity

Quantitative analysis of Rac1 and RhoA activation was performed using a G-LISA Rac1 or RhoA activation assay (Cytoskeleton, Denver, CO, USA). Briefly, cell lysates were prepared from the cell lines at determined times following plating at 50% confluence. Samples at a normalized total protein concentration of 0.5 mg/ml were used in determinations of Rac1 activity in a 96-well plate format according to the manufacturer's instructions using a microplate reader DTX880 Multimode detector (Beckman Coulter, Brea, CA, USA).

### Spreading curve

One hundred ml of complete media with 1000 cells per ml were seeded per well in 8-well slides (Labtek, Campbell, CA, USA) and F-actin stained, and mounted as described above. 40X confocal images were used to automatically determine cells areas with CellProfiler. Normalized cell areas versus 24 hours-cell area were used to estimate half-spreading time from time-adjusted hyperbolic curve where:

Normalized Area=100*Time/(Half-spreading Time+Time)

Half-spreading time was used as an estimator of average velocity of cell spreading.

### Flow cytometry analysis

Subconfluent cells were detached with 1.25 mM EDTA in PBS, washed with ice-cold medium and blocked with 10% goat or rabbit normal serum-containing PBA (PBS/5 mg/ml BSA/0.01% sodium azide). Incubations with antibodies were performed using 2 μg/ml antibody in PBA. All washes with PBA and incubations were prepared at 4°C. Cells were finally suspended in ice-cold PBS and analyzed using a FACSCalibur Flow Cytometer (Becton-Dickinson, Franklin Lakes, NJ, USA). Acquired data were analyzed using CyFlogic software (CyFlo Ltd, Turku, Finland) as in [26].

### Data analysis

Statistical significance for adhesion, spreading and migration assays were estimated by ANOVA using Dunnet or Bonferroni posttest as described in figures using Prism5 (GraphPad Software, Inc., San Diego, CA, USA). To acquire sufficient data for statistical analysis, each experiment was performed at least three times.

## Results

### SPARC controls the actin cytoskeleton architecture

**Fig 1:**
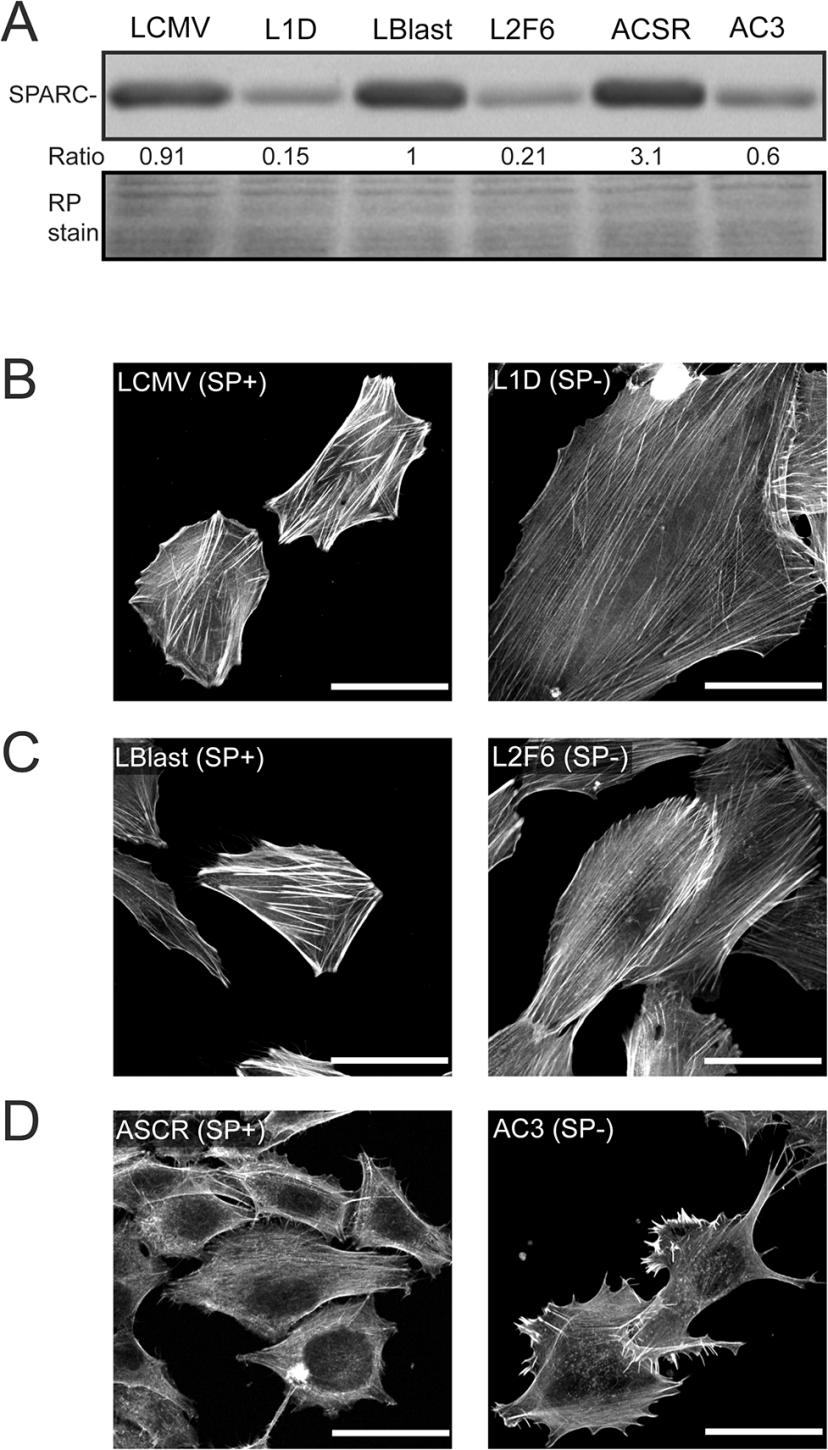
Downregulation of SPARC in melanoma cells induces actin cytoskeleton rearrangement. A) Western analysis of SPARC in the conditioned media of the different melanoma cell types. Ratio: data was normalized to the levels of SPARC expression in LBlast normalized to Ponceau S levels in each lane (RP stain). (B-D) Phalloidin staining of actin cytoskeleton. Scaling bar represents 20μm.

In order to identify the intracellular mechanisms by which SPARC might promote melanoma aggressiveness, we used established human melanoma cells where the knock down of SPARC expression was attained expressing either antisense RNA in human melanoma cells. L1D and L2F6 are SPARC-deficient cell clones obtained after transfection of IIB-MEL-LES with a SPARC-targeted antisense RNA and a shRNA, respectively [6, 27]. LCMV is the control of L1D and was transfected with an empty vector, while LBlast is the control of L2F6 and was obtained using a scramble shRNA [6]. AC3 is a SPARC-deficient cell clone obtained after transfection of A375 cells with a SPARC-targeted shRNA while control ASCR cells were obtained by transfecting parental cells with a scramble shRNA [18]. Western analysis confirmed decreased SPARC levels in the conditioned media of SPARC-deficient cells clones (SP-) compared to their respective controls (SP+)(Fig 1A).

Control cells had a cytoskeleton composed of short and thick actin stress fibers (Fig 1). In contrast, SPARC-deficient cells L1D and L2F6 exhibited expanded cell size and flattened morphology, with an actin cytoskeleton consisting of groups of long and parallel thin bundles spanning the entire cell length, ending mainly at the cell edge (Fig 1); these cells were also more resistant to detachment by trypsin treatment (not shown). Additionally, although ASCR control melanoma cells did not exhibit a highly organized actin cytoskeleton, SPARC-deficient AC3 cells showed a marked actin cytoskeleton rearrangement with increased formation of filopodial projections compared to ASCR control cells (Fig 1D).

**Fig 2:**
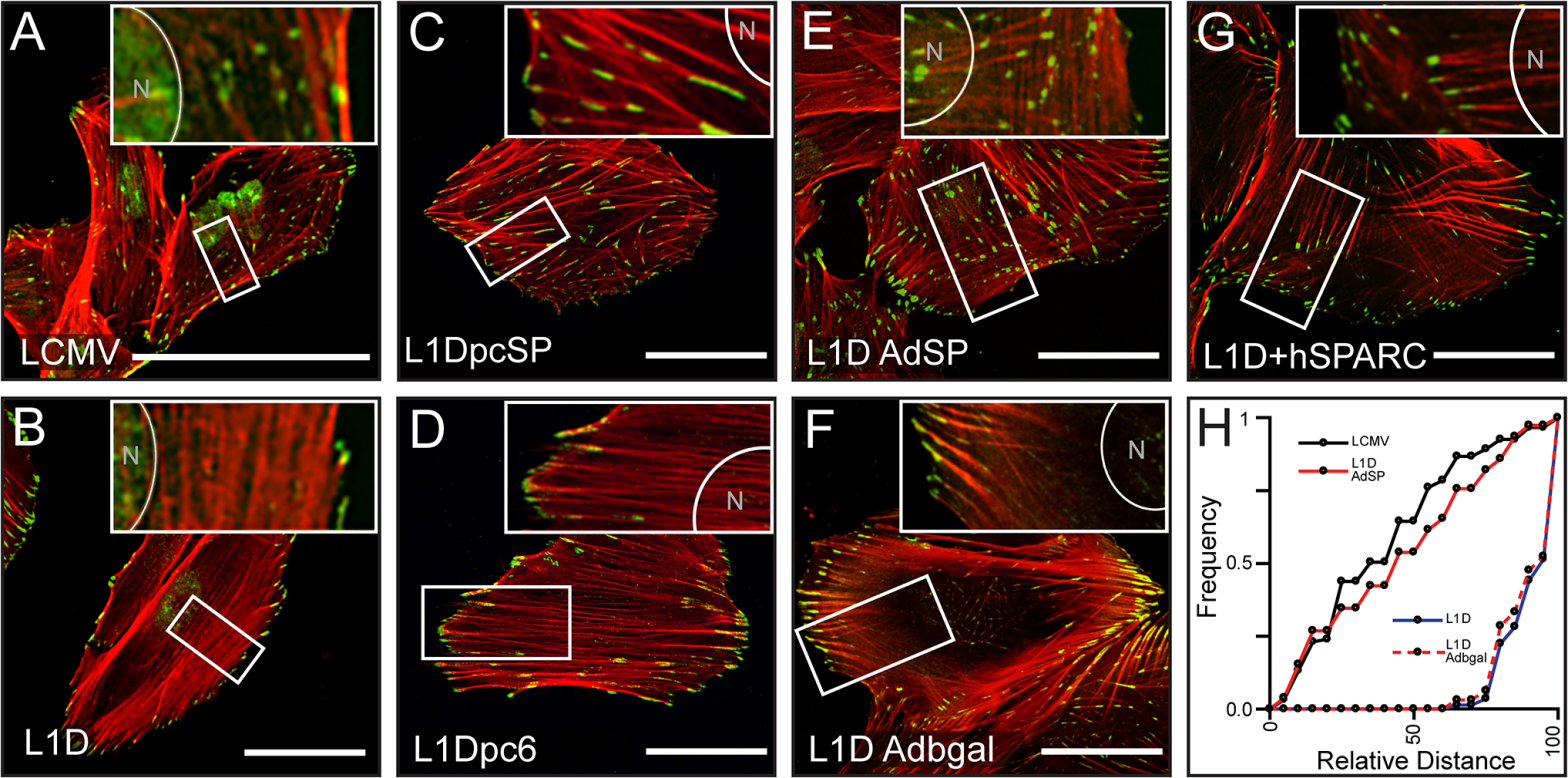
Actin cytoskeleton architecture and focal contacts localization are regulated by SPARC. Phalloidin (red) and phospho-Tyrosine (green) staining of cells plated on serum: A) LCMV control cells; B-G) SPARC-deficient L1D cells treated as follows: transfected with SPARC encoding plasmid (C); transfected with a control plasmid (D); transduced with an adenovirus expressing SPARC (E); transduced with an adenovirus expressing β-galactosidase (F); treated with 2μg/ml of hSPARC. Insets show nuclei location (N). Scaling bar represents 20μm. (H) Focal contacts distribution: is expressed as the accumulative frequencies of the relative distance of focal contacts to the center of the nucleus (N). Data was obtained from 15 to 20 cells in each treatment.

The combined staining of phalloidin and p-Tyr to identify focal contacts in LCMV control cells showed an even distribution of focal contacts at the tips of the short actin bundles (Fig 2A). In contrast, SPARC-deficient L1D cells exhibited peripheral focal contacts associated with the long actin stress fibers (Fig 2B). Restoration of SPARC expression in SPARC-deficient cells using a plasmid (Fig 2C and Fig A in S1 File. A), a viral vector (Fig 2E) or its exogenous addition (Fig 2G) induced cytoskeleton and focal adhesions rearrangement leading to a cell phenotype that resembled control LCMV cells. In contrast, transfection of SPARC-deficient L1D cells with an empty plasmid vector (Fig 2D) or transduction with an adenovirus expressing β-galactosidase (Fig 2F) had no effect on cytoskeleton organization. Quantification of the relative distance of the focal contacts from the nucleus confirmed that most of the focal contacts were localized at the periphery in SPARC-deficient cells (Fig 2H and Fig B-C in S1 File). SPARC restoration either by its re-induced expression or by its exogenous addition restored the relative distance of focal contacts in SPARC-deficient cells to the relative distance observed in control cells (Fig 2H and Fig D in S1 File). The co-localization of β1 integrin and p-Tyr with vinculin confirmed that we were assessing focal contacts (Fig E-F in S1 File).

### SPARC modulates melanoma cell adhesion to different matrices involving specific integrins

**Fig 3:**
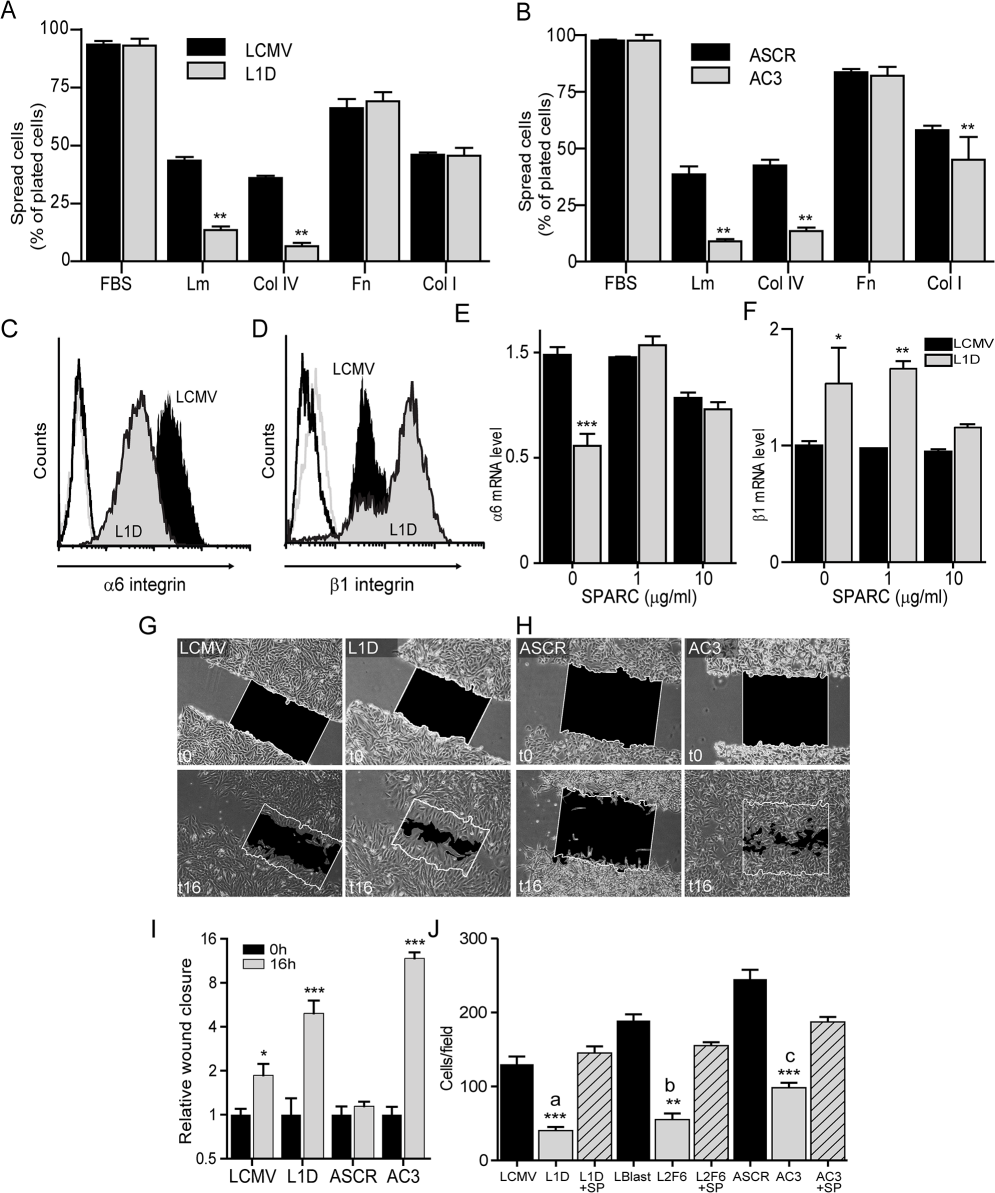
SPARC regulates cell adhesion, integrin expression and migration. A) Spreading assays on different ECM protein components of A) Mel LES- and B) A375- derived cells. Cell surface expression levels of C) α6 and (D) β1integrin. (E) α6 and (F) β1 integrin mRNA expression measured by qPCR. *p* values are from two factor ANOVA; ** p<0.01, *** p<0.001 (compared to levels in LCMV). (G-H) Motility of melanoma cells using the scratch assay (I) Cell motility expressed as the level of wound closure by each cell type compared to their respective control (n = 4 scratches). J) Migration of melanoma cells. Error bars represent average +/- S.E. from four independent experiments. *p* values are from two factor ANOVA;* p<0,05, ** p<0.01, *** p<0.001 (compared to levels in LCMV (a), to LBlast (b), or to ASCR(c).

The changes observed in cytoskeleton architecture of SPARC-deficient cells occurred in parallel to their reduced capacity to adhere to laminin and collagen type IV (Fig 3A); on the other hand, we observed no major changes in the adhesiveness of SPARC to fibronectin and collagen type I compared to control cells (Fig 3A and 3B).

In coincidence with these changes, SPARC-deficient cells also exhibited reduced cell surface expression and mRNA levels of the laminin receptor α6 integrin (Fig 3C and 3D), and increased expression of β1 integrin (Fig 3E and 3F) that re-localized to the periphery of SPARC-deficient cells associated with focal contacts (S1 File and data not shown). Other integrins such as α2, α3 and α5 showed no changes in their expression levels (data not shown). Overnight incubation of SPARC-deficient cells with SPARC restored α6 and β1mRNA expression levels while SPARC had no effect on control cells (Fig 3E and 3F).

In 2D migration on a planar substratum (scratch assay) SPARC-deficient cells exhibited extensive lamellipodial formation and augmented capacity of migration compared to control cells (Fig 3G–3I). On the contrary, SPARC-deficient cells exhibited 50% to 70% inhibition in their migration capacity in a transwell system regardless of whether fibronectin or collagen type I were used for filters coating; this inhibition could be rescued by overnight incubation with SPARC (Fig 3J).

Taken together, these data show that SPARC modulates differentially melanoma cells capacity to adhere to certain matrices affecting cells’ migration. Interestingly, SPARC-deficient cells exhibited a contrasting migration capacity when their motility was assessed in a planar 2D assay compared to the transwell system (see the Discussion section).

### SPARC modulates cell size

**Fig 4:**
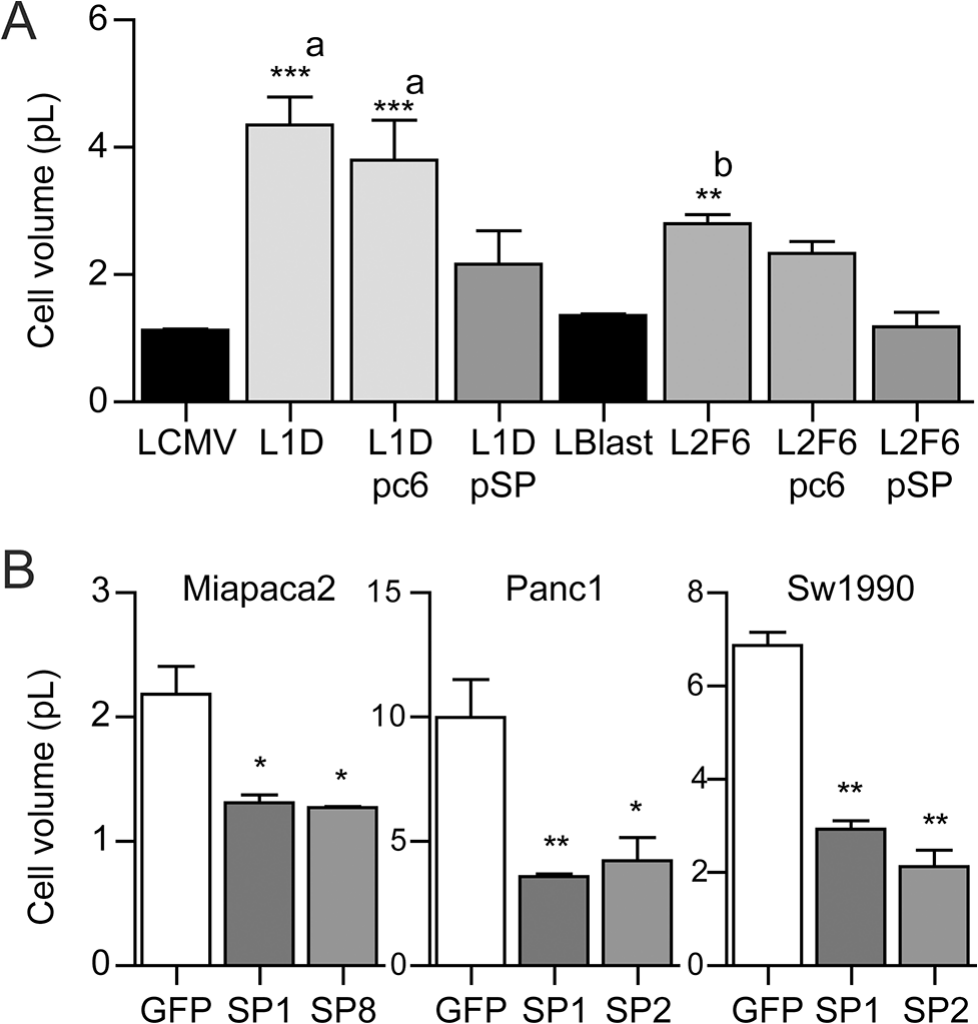
SPARC modulates cell size. Cell size of melanoma cells (A) and pancreatic cancer cells (B) expressing different levels of SPARC. Bars are the average cell size ± SE expressed in picoliters. Data was obtained from at least five independent experiments and over 300 cells analyzed. ***, **,* indicates p<0.001, p<0.01 and, p<0.05 respectively, obtained by two way ANOVA analyses. (a) the comparison refers to LCMV cells; (b) the comparison refers to LBlast.

The previous evidence indicated that SPARC-deficient cells exhibited a larger size than control cells regardless of the matrices cells were plated on. Thus, it was tempting to establish whether SPARC might indeed modulate cell size in non-attached cells. Indeed, SPARC-deficient cells exhibited 2–3 fold increase in cell size compared to their paired control cells (Fig 4A and Fig A-B in S2 File). SPARC re-expression in SPARC-deficient cells induced a clear reduction in their size to the average cell size observed in control cells (Fig 4A). Interestingly, SPARC-dependent modulation of cell size was not restricted to melanoma cells; indeed, enforced expression of SPARC in SPARC-negative pancreatic cancer cells reduced cells’ volume by 55% to 65% (Fig 4B), confirming that SPARC has the ability to control cell size at least in different malignant cell types.

**Fig 5:**
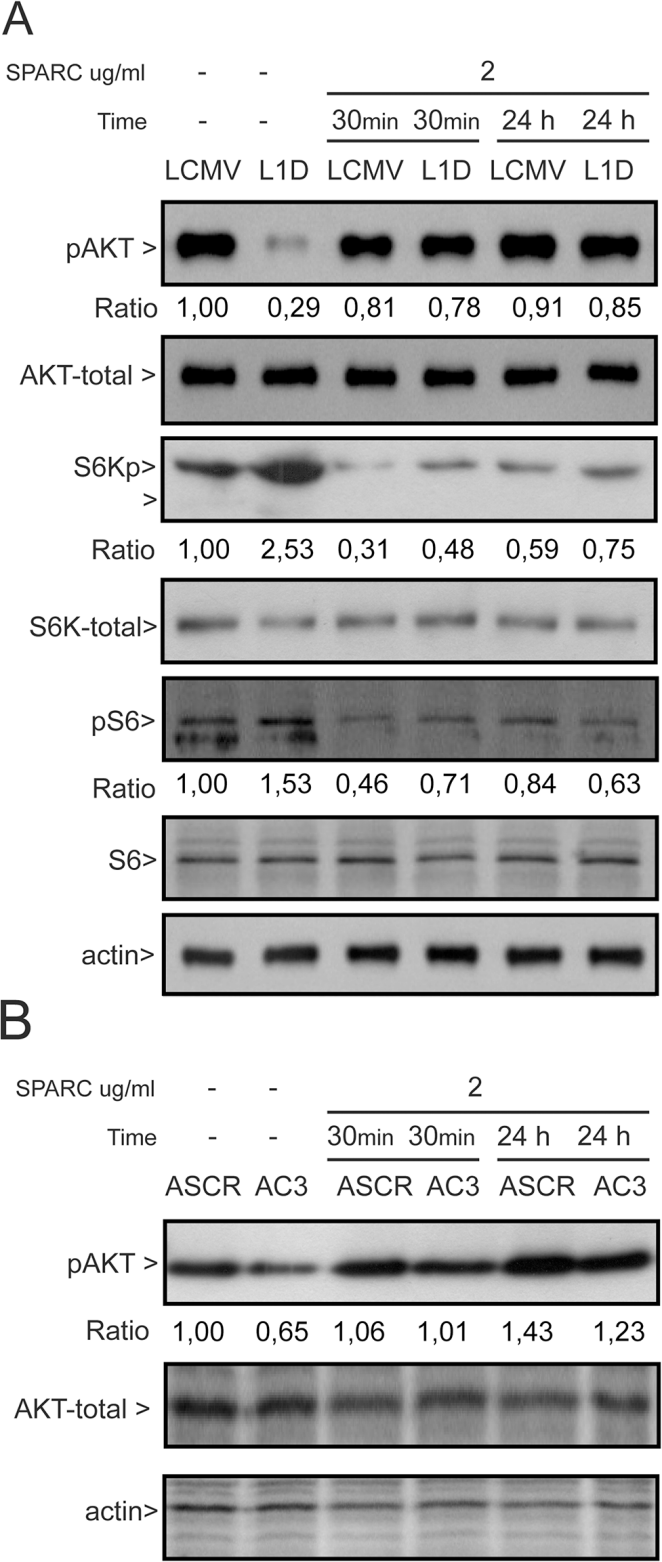
Changes in levels of activity of members of the Akt/mTOR pathway. (A) Immunoblots of phospho-Akt(ser473), Akt, phospho S6K and S6 in control and SPARC-deficient cells derived from IIB-MEL-LES cells. (B) Immunoblots of phospho-Akt(ser473) and Akt in A375 derived cells. Loading controls were assessed in separate gels.

The Akt/mTOR pathway is associated with the modulation of cell size and cytoskeleton organization [28]. On the other hand, Akt has been shown to mediate SPARC-mediated transition to a mesenchymal phenotype in melanoma cells [14]. In order to establish whether the Akt/mTOR pathway is involved in SPARC effect on cell size we assessed changes in the activity of different components of the pathway, SPARC-deficient cells exhibited reduced Ser-473 phosphorylation of Akt compared to their respective controls (Fig 5A and 5B). Phosphorylated Akt-Ser473 levels were restored in SPARC-deficient cells following 30 minutes incubation with SPARC demonstrating that Akt phosphorylation is under direct SPARC control (Fig 5A and 5B).

Previous studies have shown that Akt activation is associated with increased activity of mTORC1 and S6K [29, 30]. To our surprise, the reduced p-Akt levels observed in SPARC-deficient cells were accompanied by a significantly increased phosphorylation of S6K and its downstream target S6 showing that mTORC1 activation, and hence the increase in size of SPARC-deficient cells, occurs even in a context of reduced Akt activity (Fig 5A). Interestingly, addition of SPARC was also able to reduce the levels of S6K both in SPARC-deficient cells as in control cells (Fig 5A).

### SPARC controls Rac1 activity and intracellular localization

**Fig 6:**
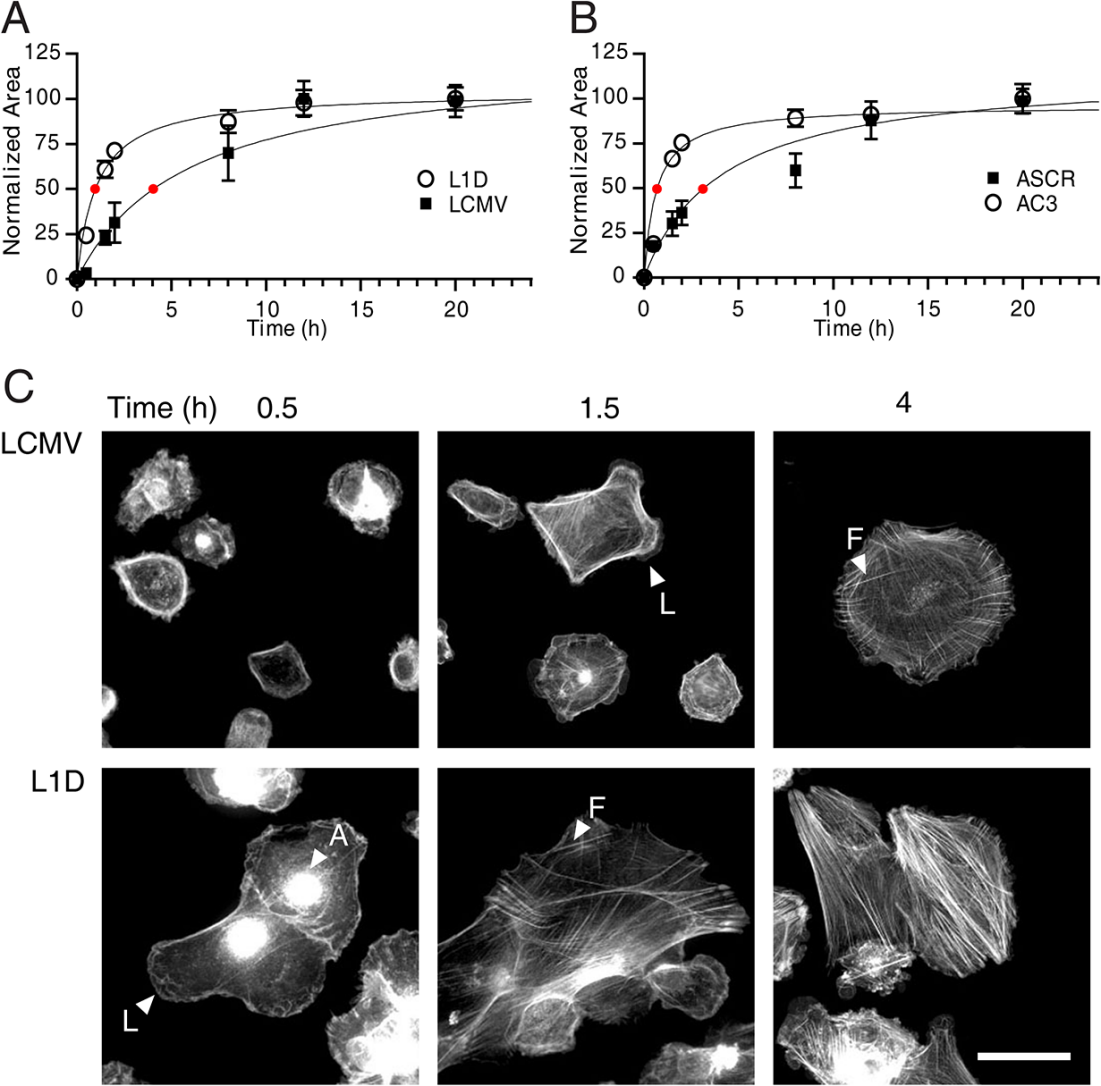
SPARC regulates melanoma cell spreading capacity. Time course of cells’ spreading. Red spot indicates half-spreading time of each cell line. C) Phalloidin staining of the different melanoma cell types at different times after seeding on FBS-coated slides. Filamentous actin (A), lamellipodia (L) and thick stress fibers (F) are indicated.

Fig 1 showed that SPARC-deficient cells not only exhibited a larger size but in some cases such as in clone AC3 also displayed abundant filopodia. In order to confirm if the enforced downregulation of SPARC expression might induce morphological changes in addition to the cytoskeleton architecture we carefully followed the cell spreading process. Spreading analysis showed that control LCMV and ASCR cells have a half spreading-time of 5.8 and 4.4 hours, while L1D and AC3 SPARC-deficient cells have a half spreading-time of 1.4 and 1.1hours, respectively. Spreading SPARC-deficient cells exhibited large lamellipodia as soon as 30 minutes after seeding (Fig 6) confirming that SPARC-deficient cells showed an augmented capacity of lamellipodial extension.

**Fig 7:**
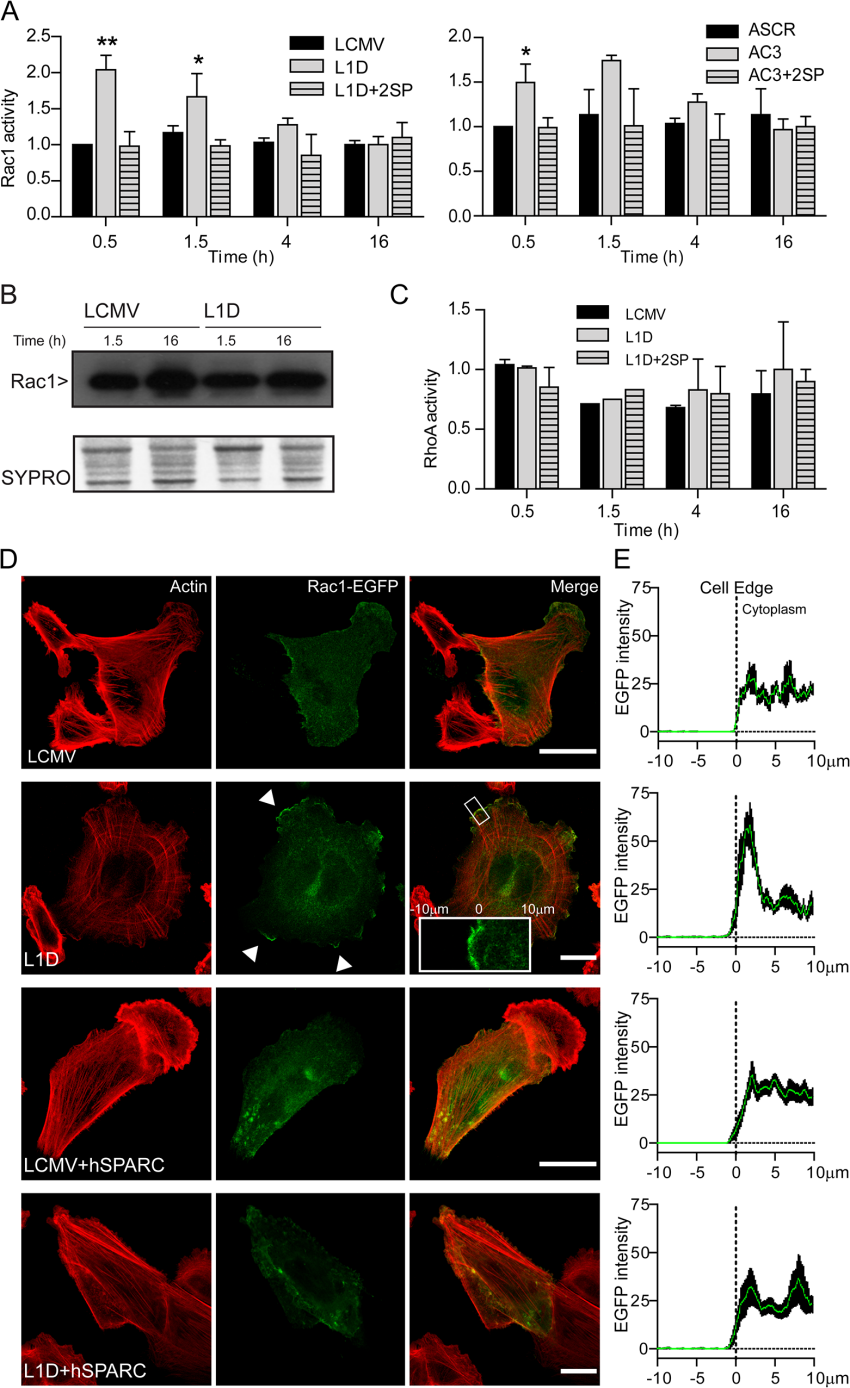
SPARC regulates the activity and localization of Rac1. (A) Quantification of Rac1-GTP analyzed by G-LISA kit in the different cell types. (B) Rac1 immunoblots in the different cell types. (C) Quantification of RhoA-GTP analyzed by G-LISA kit in the different cell types. (D) Phalloidin staining (red) of control LCMV or EGFP signal in SPARC deficient L1D cells transfected with Rac1-EGFP. White-arrows indicate lamellipodial accumulation of Rac1-EGFP. Scaling bars represent 25um. Inset: Crop of 20μm square used to measure EGFP intensity. E) Plot of Rac1-EGFP median intensity (green line) ± SD (black line) measured around 10μm of cell edge in at least 20 cells in the presence or not of 2μg/ml SPARC.

The GTPase Rac1 is responsible for lamellipodia formation and has been shown to regulate cell size, spreading and actin cytoskeleton organization [31–33]. Accordingly, we hypothesized that Rac1 could be a likely candidate as a mediator of the changes triggered by SPARC. Assessment of Rac1 activity by quantification of Rac1-GTP levels, revealed 50% to 100% increased Rac1 activity in spread SPARC-deficient cells compared to control cells (Fig 7A); no changes were observed in Rac1 total protein levels (Fig 7B). Interestingly, exogenous addition of SPARC to SPARC-deficient L1D and AC3 cells reduced Rac1 activity to the levels observed in control cells (Fig 7A).

This effect occurred as soon as 30 minutes after SPARC addition indicating that SPARC directly modulates Rac1-GTP levels. Of note, SPARC addition had no effect on control cells clearly suggesting that the changes observed in Rac1 levels are linked to the presence of SPARC (data not shown). Since these differences could be due at least in part to mutated hyper-activated Rac1 [34, 35], we sequenced Rac1 mRNA in our melanoma cells. Sequencing of Rac1 mRNA expressed either by control or SPARC-deficient cells revealed no mutation, thereby indicating that these cells expressed the wild type version of Rac1 (data not shown). Rho and cdc42 are additional members of the small GTPase family[36]. As RhoA has been also implicated in the modulation of Rac1 activity, we assessed the levels of RhoA-GTP; the data clearly showed no difference between control cells and SPARC-deficient cells (Fig 7C) indicating that SPARC does not affect RhoA activity and hence RhoA does not mediate SPARC effects on Rac1 activity.

Full activation of Rac1 is reached when the sGTPase is localized at the cell membrane, where it promotes the development of lamellipodia and ruffles [33, 37]. To establish whether SPARC might affect the intracellular localization of Rac1, we made use of the chimera Rac1-eGFP that was expressed in control and SPARC-deficient cells. LCMV control cells showed an evenly distributed eGFP signal in the cytoplasm with slight lamellipodial accumulation (Fig 7D and 7E). Interestingly, spread SPARC-deficient cells demonstrated higher eGFP signal in lamellipodia (Fig 7D) that was consistent with the peak of eGFP activity observed at the cell membrane (Fig 7E). Treatment of SPARC-deficient cells with hSPARC restored the even distribution of eGFP signal observed in control cells that is consistent with the delocalization of Rac1 from the plasma membrane (Fig 7D and 7E).

### Blockade of Rac1 reverts most of the changes observed in SPARC-deficient cells

**Fig 8:**
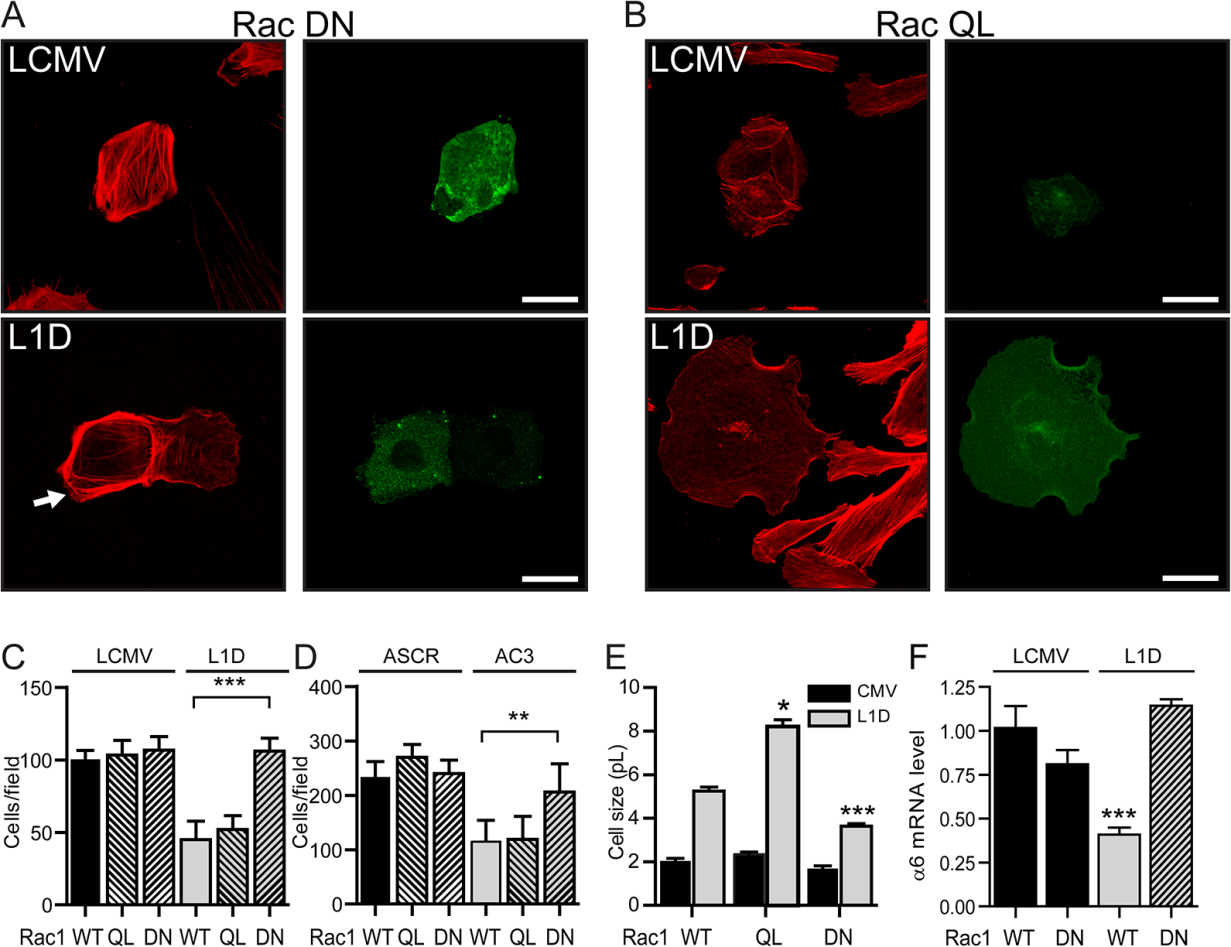
Blockade of Rac1 activity reverts the changes induced by the enforced downregulation of SPARC expression. (A) Phalloidin staining (red) of cells transfected with RacDN or (B) RacQL expressing eGFP (green) after 90min of spreading. C, D) Migration of the different melanoma cell types. (E) Cell size of cells transfected with Rac1 mutants. F) α6 integrin mRNA levels. Bars shows mean ± SE of three experiments (two-way ANOVA with Dunnet posttest, ***, ** indicates p<0.001, p<0.01 respectively).

The previous data suggested that SPARC regulates Rac1 activity as well as its intracellular localization. To establish whether Rac1 might mediate SPARC effects on melanoma cell plasticity, we made use of a constitutively active Rac1 mutant (RacQL) and a dominant negative Rac1 mutant (RacDN) to modulate Rac1 activity. Before starting a large series of experiments with Rac1 mutants we performed preliminary studies to ascertain whether RhoA and Cdc42 are involved in this process. For this purpose we ran parallel experiments with the mutant forms of all the three members of the GTPase family. The mutant forms of RhoA and Cdc42 exerted the expected effects on cytoskeleton architecture both on control LCMV cells as in SPARC-deficient L1D cells. Hence, these effects were independent of SPARC levels. On the contrary, expressing RacDN, and in some cases RacQL, in SPARC-deficient cells had a dramatic effect on their cell cytoskeleton which completely differs from the effect observed in control LCMV cells. In fact, expression of RacQL or RacDN had no apparent effect on LCMV actin cytoskeleton. In contrast, close to 100% of RacDN-transfected SPARC-deficient cells showed a rearranged actin cytoskeleton with actin fibers located at the cell periphery (Fig 8A); in addition, almost 40% of RacQL-transfected SPARC-deficient cells showed prominent lamellipodia confirming that Rac1 can actively modulate cell morphology only in cells where SPARC expression goes down below a certain threshold (Fig 8B).

We next assessed whether blockade of Rac1 activity by expressing RacDN can restore the changes in cell behavior induced by downregulation of SPARC expression. Interestingly, expression of RacDN, but not RacQL, in SPARC-deficient cells restored cells migration through transwells to the levels of control cells (Fig 8C and 8D). As expected from the previous data, RacDN and RacQL had no effect on control cells (Fig 8C and 8D). On the other hand, overexpression of RacQL induced a significant increase in the cell size of SPARC-deficient cells L1D and L2F6 (Fig 8E and Fig B in S3 File); while RacDN induced only a slight reduction in the size of SPARC-deficient L1D cells (Fig 8E) and was unable to affect L2F6 cells’ size (Fig B in S3 File). Neither the expression of RacQL nor of RacDN affected the cell size of control melanoma cells (Fig 8E and Fig B in S3 File). Interestingly, RacDN expression in SPARC-deficient cells was able to restore α6 (Fig 8F) but not β1 integrin levels (Fig C in S3 File) to those observed in control cells suggesting that α6 is a direct target of SPARC while the changes in β1 integrin levels and localization are probably secondary to the changes in cytoskeleton architecture induced by SPARC.

## Discussion

The present data shed light on a potential mechanism by which SPARC might promote an aggressive phenotype by demonstrating that this transition involves Rac1. The present data indicates that SPARC is a permissive factor for Rac1 that becomes fully active only when SPARC levels are reduced below a certain threshold. SPARC modulates both Rac1-GTP levels and Rac1 transition from an active membrane bound form to a cytoplasm localized-inactive protein. Exposing SPARC-deficient cells to a dominant negative form of Rac1 promoted cells’ transition to a more aggressive phenotype that resembled control melanoma cells.

It was unexpected that the modulation by SPARC of the transition to a more aggressive mesenchymal phenotype, and the reversion back, involved a dramatic change in the size of melanoma and pancreatic cancer cells. An increase in cell size was associated with a delay in the G1/S phase [38, 39]; interestingly, SPARC-deficient melanoma cells exhibited a delay in S phase entry suggesting a clear link between the downregulation of SPARC levels, increase in cells’ size and cycling arrest [3, 40–42]. Moreover, metastatic melanoma cells and migrating glioblastoma cells also showed reduced cell size compared to their respective controls [43, 44]. The question as to whether a SPARC-mediated reduction in cell size is a *sine qua non* requirement for a malignant cell to exit G0 and avoid cycling to start migration and disseminate warrants further studies.

The evidence that the suppression of SPARC expression induced the formation of lamellipodia extensions led us to pursue the possibility that Rac1 could be the hub connecting all the aggressive features induced by SPARC. Less aggressive SPARC-deficient cells exhibited increased levels of Rac1-GTP concomitant with Rac1 translocation to the cell membrane; re-localization of Rac1 at the cell membrane was associated with a less aggressive phenotype that included a dramatic increase in cell size of SPARC-deficient cells and the activation of mTORC1 as demonstrated by the increased levels of phosphorylated pS6K and pS6. SPARC was able to modulate S6K phosphorylation immediately after 30 minutes, suggesting that SPARC-induced changes in cell size are mediated by mTORC1 and are under direct control of SPARC. Of note, SPARC-deficient cells showed increased motility in a 2-D planar assay consistent with Rac1 activation and extension of large lamellipodia; on the contrary, SPARC-deficient cells showed reduced capacity to migrate through a non-planar more complex transwell system that requires a relaxed or plastic cell cytoskeleton; therefore SPARC-deficient cells exhibiting increased Rac1 activity might have reduced capacity to migrate through transwells due to increased cytoskeleton stiffness. This is consistent with the abrogated in vivo tumorigenic capacity of SPARC-deficient melanoma cells [3, 5].

Of note was that RacQL was able to induce a further increase in the cell size of SPARC-deficient cells while RacDN had no effect. On the contrary, a previous study with normal cells has shown that binding of Rac1 to membrane-bound mTORC is independent of its GTP loading [33]. In this regard it was unexpected that SPARC-deficient cells exhibited reduced levels of pAkt a downstream target of mTORC2, suggesting that SPARC-deficient cells have reduced mTORC2 activity compared to control cells [45, 46]. Since reduced pAkt levels occurred in parallel to the mobilization of Rac1 to the cell membrane, it is likely that Rac1-GTP binding to the cell membrane may shift the balance to the formation of mTORC1 through the inhibition of mTORC2 formation. The question whether the difference in Rac1 binding capacity to mTOR complex 1 or 2 is due to a transition to a malignant phenotype remains open.

The immediacy of these effects on Akt/mTORC indicates that SPARC might have a direct link to this pathway via a cell surface receptor. It has been postulated that α5β1 [47] and stabilin [48] might act as SPARC receptors. Efforts from different groups including ours failed in their aim to find a cognate receptor. This might arise in part from the fact that SPARC is a member of the naturally unfolded proteins [49] and as such might interact with different extracellular and cell surface proteins simultaneously exerting complex downstream effects.

Despite the immediate effect of SPARC on the modulation of the Akt/mTORC pathway reversion of cells´ phenotype required overnight incubation. Moreover, we were unable to see any direct interaction between SPARC and Rac1 either by co-immunoprecitation or co-localization. Thus, it seems that SPARC is triggering intracellular processes that modulate Rac1 transition from the membrane to the cytoplasm and back. SPARC can regulate melanoma cell transition to an aggressive mesenchymal phenotype (that implies Rac1-GDP localization at the cytoplasm) by inducing the shift from E- to N-cadherin expression [6, 50]. Interestingly, mutant B-Raf regulates a shift to N-cadherin and enhanced invasiveness of melanoma cells through the downregulation of Rac1 activity [51]. In addition, α6β1that was shown to promote the metastatic phenotype of melanoma cells [52, 53] is transcriptionally regulated by TGFβ that interacts with SPARC through reciprocal activation [2]; Thus, it is tempting to hypothesize that a link might exist between SPARC, B-Raf, α6 integrin and N-cadherin that inactivates Rac1. Consistent with this hypothesis, the involvement of E-cadherin in cell adherence events requires Rac1 [37], and blocking E-cadherin with specific antibodies downregulates Rac1 activity [54]. The potential pathway linking SPARC with Rac1 activity and localization could involve the downregulation of E-cadherin expression by SPARC, thereby leading to the downregulation of Rac1 activity and hence stimulating the transition to a mesenchymal phenotype.

## Supporting Information

S1 File SPARC modulates focal contact distribution (FC). (Fig A) Western analysis of SPARC expression in control LCMV cells and SPARC-deficient cells L1D transfected with SPARC expressing plasmids or adenovirus and their respective controls. Loading control and ratio were calculated as in Fig 1A. (Fig B-C) Phospho-tyrosine staining of LCMV control cells (B) and SPARC-deficient cells L1D (Fig C). (Fig D) Distribution of relative distances of focal contacts in control and SPARC-deficient L1D cells transfected with a plasmid encoding SPARC, empty plasmid or treated with SPARC. (Fig E) β1 and (Fig F) p-Tyr co-stained with vinculin in SPARC-deficient cells L1D. Insets are magnification of the indicated area. Scaling bars represent 20μm.
(PDF)

S2 File SPARC modulates cell size. SPARC modulates cell size. For Fig A and Fig B see more details in Fig 3. (two-way ANOVA with Dunnet posttest, ***, ** indicates p<0.001, p<0.01 respectively; (a) data refers to LCMV, (b) to LBlast, or (c) to ASCR.
(PDF)

S3 File RacDN and RacQL, cell size and integrin levels. (Fig A) Control ASCR and SPARC-deficient AC3 cells transfected with wild-type and mutant versions of Rac1. After 24h whole cell lysate was subjected to Western blot analysis with anti-phospho S473-Akt.(Fig B) Cell volume of cells transfected with Rac1 mutants.(Fig C) β1 integrin mRNA levels. Bars shows mean ±SE of three experiments.
(PDF)
