# Supplementary figures and images for: SPARC Controls Melanoma Cell Plasticity through Rac1
Source: PLoS One. 2015 Aug 6;10(8):e0134714. doi: 10.1371/journal.pone.0134714 (PMC4527691; doi:10.1371/journal.pone.0134714)

# Figure S1

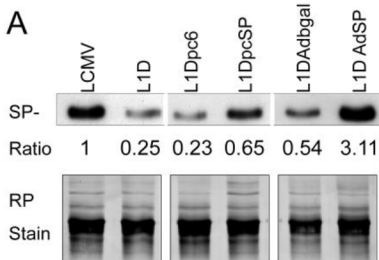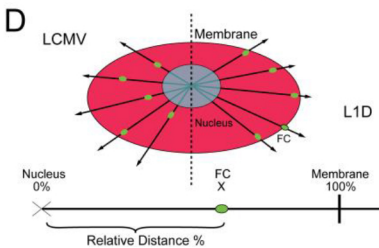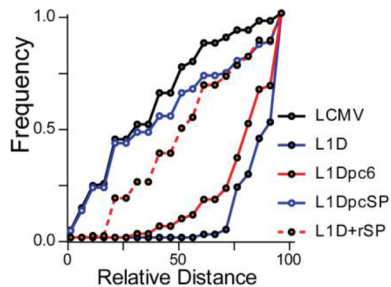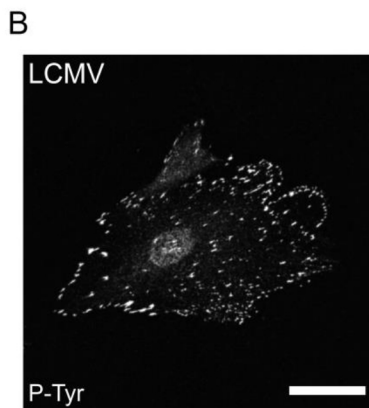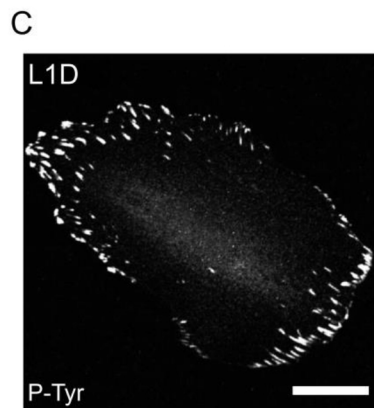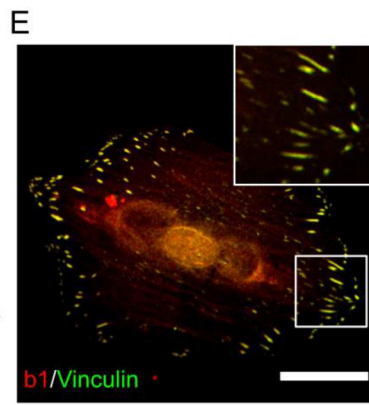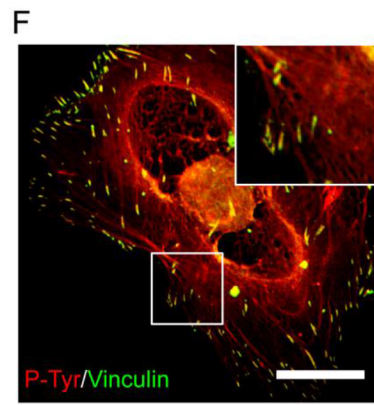

Supplement: S1 File — (Fig A) Western analysis of SPARC expression in control LCMV cells and SPARC-deficient cells L1D transfected with SPARC expressing plasmids or adenovirus and their respective controls. Loading control and ratio were calculated as in Fig 1A. (Fig B-C) Phospho-tyrosine staining of LCMV control cells (B) and SPARC-deficient cells L1D (Fig C). (Fig D) Distribution of relative distances of focal contacts in control and SPARC-deficient L1D cells transfected with a plasmid encoding SPARC, empty plasmid or treated with SPARC. (Fig E) β1 and (Fig F) p-Tyr co-stained with vinculin in SPARC-deficient cells L1D. Insets are magnification of the indicated area. Scaling bars represent 20μm. (PDF) [file pone.0134714.s001.pdf]

# Figure S2

## A

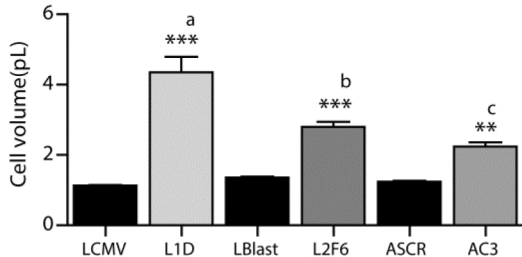

## B

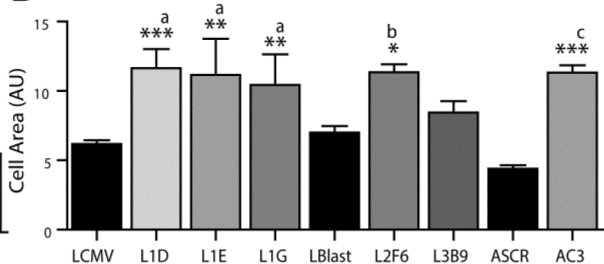

Supplement: S2 File — SPARC modulates cell size. For Fig A and Fig B see more details in Fig 3. (two-way ANOVA with Dunnet posttest, ***, ** indicates p<0.001, p<0.01 respectively; (a) data refers to LCMV, (b) to LBlast, or (c) to ASCR. (PDF) [file pone.0134714.s002.pdf]

# Figure S3

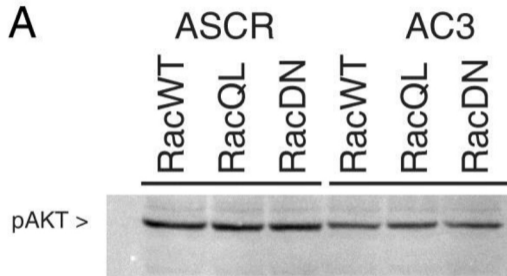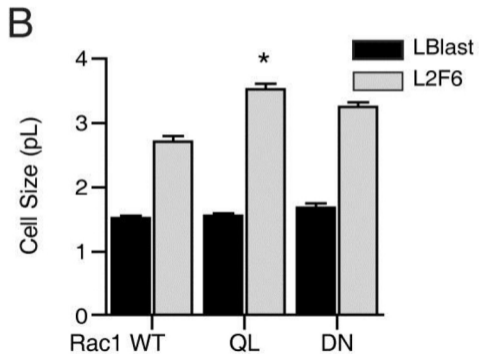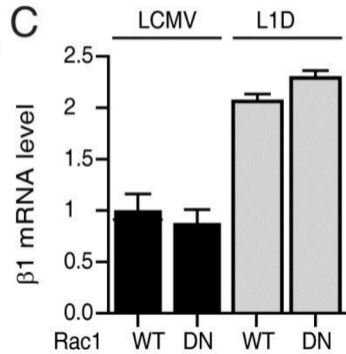

Supplement: S3 File — (Fig A) Control ASCR and SPARC-deficient AC3 cells transfected with wild-type and mutant versions of Rac1. After 24h whole cell lysate was subjected to Western blot analysis with anti-phospho S473-Akt.(Fig B) Cell volume of cells transfected with Rac1 mutants.(Fig C) β1 integrin mRNA levels. Bars shows mean ±SE of three experiments. (PDF) [file pone.0134714.s003.pdf]
